# LncPheDB: a genome-wide lncRNAs regulated phenotypes database in plants

**DOI:** 10.1007/s42994-022-00084-3

**Published:** 2022-10-05

**Authors:** Danjing Lou, Fei Li, Jinyue Ge, Weiya Fan, Ziran Liu, Yanyan Wang, Jingfen Huang, Meng Xing, Wenlong Guo, Shizhuang Wang, Weihua Qiao, Zhenyun Han, Qian Qian, Qingwen Yang, Xiaoming Zheng

**Affiliations:** 1 National Key Facility for Crop Gene Resources and Genetic Improvement, Institute of Crop Sciences, Chinese Academy of Agricultural Sciences, Beijing, 100081 China; 2 College of Life Science, Shenyang Normal University, Shenyang, 110034 China; 3 National Nanfan Research Institute (Sanya), Chinese Academy of Agricultural Sciences, Sanya, 572000 China; 4 State Key Laboratory of Rice Biology, China National Rice Research Institute, Chinese Academy of Agricultural Sciences, Hangzhou, 310006 China; 5 Agricultural Genomics Institute at Shenzhen, Chinese Academy of Agricultural Sciences, Shenzhen, 518120 China; 6 International Rice Research Institute, DAPO box 7777 Metro Manila, The Philippines

**Keywords:** LncRNA, GWAS, Phenotype, SNP, Plants

## Abstract

LncPheDB (https://www.lncphedb.com/) is a systematic resource of genome-wide long non-coding RNAs (lncRNAs)-phenotypes associations for multiple species. It was established to display the genome-wide lncRNA annotations, target genes prediction, variant-trait associations, gene-phenotype correlations, lncRNA-phenotype correlations, and the similar non-coding regions of the queried sequence in multiple species. LncPheDB sorted out a total of 203,391 lncRNA sequences, 2000 phenotypes, and 120,271 variants of nine species (*Zea mays* L., *Gossypium barbadense* L., *Triticum aestivum* L., *Lycopersicon esculentum* Mille, *Oryza sativa* L., *Hordeum vulgare* L., *Sorghum bicolor* L., *Glycine max* L., and *Cucumis sativus* L.). By exploring the relationship between lncRNAs and the genomic position of variants in genome-wide association analysis, a total of 68,862 lncRNAs were found to be related to the diversity of agronomic traits. More importantly, to facilitate the study of the functions of lncRNAs, we analyzed the possible target genes of lncRNAs, constructed a blast tool for performing similar fragmentation studies in all species, linked the pages of phenotypic studies related to lncRNAs that possess similar fragments and constructed their regulatory networks. In addition, LncPheDB also provides a user-friendly interface, a genome visualization platform, and multi-level and multi-modal convenient data search engine. We believe that LncPheDB plays a crucial role in mining lncRNA-related plant data.

**Supplementary Information:**

The online version contains supplementary material available at 10.1007/s42994-022-00084-3.

## Introduction

LncRNAs are a class of non-coding RNAs that are more than 200 nucleotides in length. Initially, this type of RNA was once considered to be “junk” material in the genome. However, as the research continues, there is growing evidence that lncRNAs are key players in growth and development, metabolism and regulatory processes in a variety of organisms, particularly in mammals and humans (Kopp and Mendell 2018; Kung et al. 2013; Morris and Mattick 2014; Sun et al. 2018; Uchida and Dimmeler 2015; Wu et al. 2017). However, the study of lncRNAs in plants remains in its infancy. Currently, it has been found in plants that lncRNAs not only play an important role in regulating growth and developmental processes such as growth hormone transport and signal transduction in plants. It also plays an important role in improving crop yield (Wang et al. 2018)**,** leaf distortion (Liu et al. 2018)**,** plant fertility (Fang et al. 2019; Zhao et al. 2018), fruit fertility (Fan et al. 2016) and other important agronomic traits. But the vast majority of lncRNA regulatory explorations with clear mechanisms are nowadays performed in *Arabidopsis thaliana*. Our understanding of the mechanisms regulating lncRNAs in crop species remains limited. In addition, in recent years, transcriptome data have been used to carry out a large number of lncRNAs-related studies (Katayama et al. 2005; Osato et al. 2003; Terryn and Rouzé 2000; Wang et al. 2005; Zhang et al. 2006, 2014; Zhu and Deng 2012). Studies have shown that there are 32,397 lncRNAs in maize, 11,565 lncRNAs in rice, and 12,577 lncRNAs in soybean (Jin et al. 2021). It has also been revealed that lncRNAs are generally characterized by low expression, poor conservativeness among different species, and tissue specificity (Derrien et al. 2012; Cabili et al. 2011). These characteristics make the study of lncRNAs functions a herculean task. At present, although a large number of lncRNAs have been identified through transcriptome research, the lncRNAs whose functions have been further verified are less than 1% (Quek et al. 2015). Furthermore, the genome-wide association study (GWAS) of multiple species revealed that 84% of trait-related variation loci are located in non-coding sequences (Cheetham et al. 2013). However, the non-coding regions in the genome lack annotations and other relevant information. This hinders our further research on the non-coding regions.

The lncRNAs database is a very good tool to facilitate a detailed and accurate study of lncRNAs. In recent years, a total of 20 plant-related lncRNA databases have been established. They have averaged a whopping 530 citations since publication. But most of these databases provide the basic information of lncRNAs in species and target gene prediction according to transcriptome data. For instance, the PLncDB database (Jin et al. 2021) can provide basic information about various plants, such as lncRNA genome position, sequence, and structure, the expression in tissues, and the query and visual display of gene regulation networks. However, the database can only perform a Basic Local Alignment Search Tool (BLAST) analysis of single species. The CANTATAdb 2.0 database (Szcześniak et al. 2019), which contains lncRNAs of plants and algae, leverages on JBrowse, eFP Browser, EPexplorer, and other analysis tools to search for the maximum peptide length, maximum expression level, number of lncRNA exons, and other information of lncRNAs in species. The GreeNC database (Gallart et al. 2016) can extract the position, sequence, coding potential, folding energy, and other information of lncRNAs in various species; it can be used to perform a BLAST analysis of one or more species. Most of the databases constructed by researchers in the early days focused on some basic annotation information about the sequence and position of lncRNAs. However, they lacked comprehensive annotation information. In addition, very few databases could provide information about the correlation between lncRNAs and phenotypes, the similarity of lncRNAs among multiple species and display the possible correlation between these similar fragments and phenotypes. The RiceLncPedia database (Zhang et al. 2021), a newly built database, has comprehensive annotation information of lncRNAs. For instance, the database collects multi-omics information, such as quantitative trait locus, GWAS, transposons, and variant sites (SNPs). However, it only shows the lncRNAs of rice, but no blast tool is available to study the similarity of lncRNAs among different species. Therefore, it is necessary to build a database that explores the similarity of lncRNAs in multiple species and combines lncRNAs with GWAS.

In this study, we built a database containing the lncRNAs information of nine common crops, including *Zea mays* L., *Gossypium barbadense* L., *Triticum aestivum* L., *Lycopersicon esculentum* Mille, *Oryza sativa* L., *Hordeum vulgare* L., *Sorghum bicolor* L., *Glycine max* L., and *Cucumis sativus* L. The database provides information about the sequence and position of lncRNAs, the distribution of lncRNAs in the genome, the population variation of lncRNAs, and the phenotypic traits that may be regulated, among others. In addition, the database can also use the BLAST tool to investigate the conservativeness of target gene sequences in various species and the phenotypic conditions that may be regulated. Our database is designed to further improve the annotation information of lncRNAs in plants to further explore the possible functions of lncRNAs.

## Materials and methods

### Data collection and sorting

**Fig. 1:**
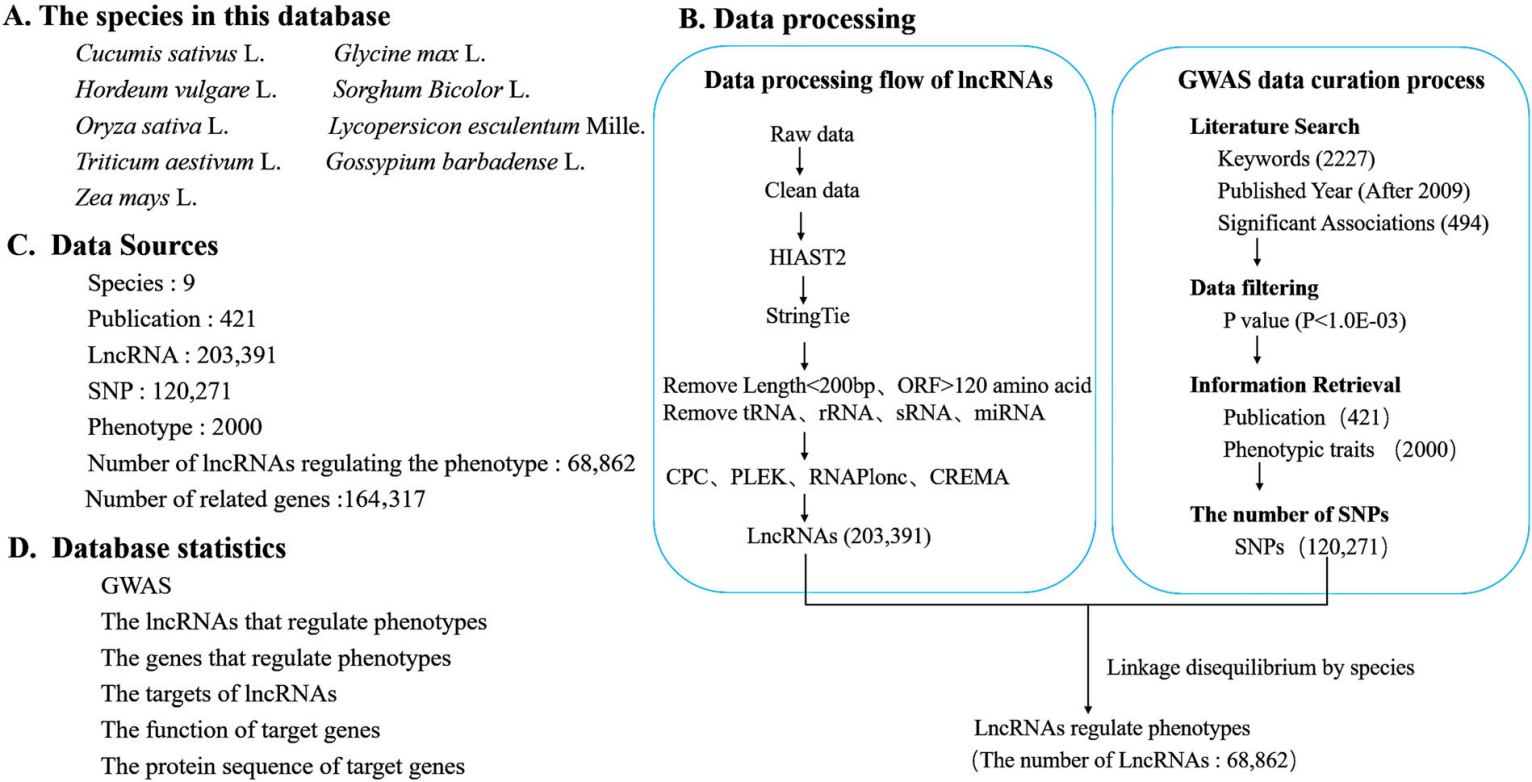
Data processing workflow and outcomes of LncPheDB. **A** The nine species included in the database. **B** The data processing workflow of lncRNA and the curation process adopted by the GWAS is on the right. **C** Summary of the data contained in LncPheDB. **D** Database statistics in this study

For the LncPheDB database, we selected nine important model plants (including *Zea mays* L., *Gossypium barbadense* L., *Triticum aestivum* L., *Lycopersicon esculentum* Mille, *Oryza sativa* L., *Hordeum vulgare* L., *Sorghum bicolor* L., *Glycine max* L., and *Cucumis sativus* L.) with great economic value and a high-quality reference genome. According to the data sequencing method and data sequencing depth, we extracted a total of 2324 RNA sequencing (RNA-Seq) datasets from the National Center for Biotechnology Information (NCBI) Sequence Read Archive (SRA) database (https://www.ncbi.nlm.nih.gov/sra/) (Supplemental Table S1). Using the SRA toolkit (Version 2.8) under the Linux system, we first converted the extracted SRA file into Fastq format and trimmed the adapter sequences using Trim Galore (version 0.50) (https://www.bioinformatics.babraham.ac.uk/projects/trim galore/) to obtain clean data. HIAST2 (Kim et al. 2015) was used to make a comparison between the clean data and the reference genome; afterward, the clean data were assembled with StringTie (Pertea et al. 2015). StringTie-merge was used to obtain the transcript set of each species. The transcripts were filtered out according to the following criteria: transcript length less than 200 base pairs and open reading frame greater than 120 amino acids. Finally, BLASTx was used to search the SWISS-PROT database to filtered out the transcripts that may encode small peptides with the parameters -e 1.0e-4-S 1. A comparison between the database and the Rfam database was performed to filter out tRNAs, rRNAs, sRNAs, and miRNAs. The transcripts were collected after the filtering. The CPC (Kong et al. 2007), CREMA (Simopoulos et al. 2018), PLEK (Li et al. 2014), and RNAplonc (Negri et al. 2019) programs were used to calculate the protein-coding ability of transcripts, and the non-protein-coding transcripts detected in at least two software were used as candidate lncRNAs (Fig. 1B). In addition, to enrich lncRNAs types, we sorted out the lncRNAs sequences of the nine species mentioned above in the RNAcentral Database (The et al. 2017) and the EVLncRNAs Databases (Zhou et al. 2018).

To extract comprehensive and high-quality information from published GWAS articles, we used the keywords “species” and “GWAS” to search for articles published in PubMed and we obtained 2227 relevant research articles that were published after 2009. Afterward, Articles were selected if there were a large number of candidates for significant SNP-phenotype correlation analysis data, while articles with segmental and phenotypic correlation data or no SNP-phenotype correlation analysis data were removed. We found 497 articles with data that are significantly related to genome-wide variation loci and phenotypic traits. Finally, 421 articles were further screened according to the *P*-value (*P* < 10^–3^) of significant GWAS data. In addition, the basic information of these articles is listed in Supplemental Table S2.

To link the lncRNAs data with the GWAS result data, we used the BWA tool (version 0.7.17) to unify the SNPs from GWAS data in each species and the reference genome from lncRNAs data in the same species into the same reference genome. Afterward, we first mapped the long segments according to the distance between SNPs (The distance between variant sites was shorter than the length of the region of linkage disequilibrium (LD)) (Supplemental Table S3), and then amplified the mapped long segments according to the LD of each species, if the lncRNAs and genes are within the incremental region, these lncRNAs are considered to regulate the corresponding phenotype and are associated with genes. At the same time, we also amplified a single site in the GWAS results based on the length of the region of LD of each species, and based on the positional relationship between the gene or lncRNA and the amplified segment, to determine the phenotypes that lncRNAs or genes may regulate (Guttman and Rinn 2012; Guttman et al. 2011; Huarte et al. 2010; Lee 2009; Martianov et al. 2007; Nagano et al. 2008; Rinn and Chang 2012; Sleutels et al. 2002).

### Implementation

LncPheDB was implemented using PostgreSQL (https://www.postgresql.org; a powerful, open-source object-relational database system with over 30 years of active development that has earned it a strong reputation for reliability, feature robustness, and performance) and Django development server (https://docs.djangoproject.com/en/2.2/intro/tutorial01/#the-development-server; a lightweight web server written purely in Python). Web user interfaces were developed using Django (https://www.djangoproject.com; a high-level Python web framework that encourages rapid development and clean, pragmatic design), HTML5, CSS3, AJAX (Asynchronous JavaScript and XML; a set of web development techniques used to create asynchronous applications without interfering with the display and behavior of the existing page), JQuery (a cross-platform and feature-rich JavaScript library; http://jquery.com, version 1.10.2), Vue (https://vuejs.org; the Progressive JavaScript Framework, version 2.6.14), layui (https://github.com/sentsin/layui/; a classic modular front-end UI framework), and Boot-Strap (an open-source toolkit for developing web projects with HTML, CSS, and JS; https://getbootstrap.com, version4.6.0). For dynamic genome visualization and analysis, JBrowse Genome Browser (a fast, scalable genome browser built completely with JavaScript and HTML5; https://jbrowse.org/jbrowse1.html, version 1.16.11) was adopted to generate interactive charts.

## Results

GWAS revealed many genetic variants associated with phenotypes. Thousands of GWAS studies have revealed that 93% of common genetic variants associated with specific traits or diseases are located in non-coding regions (Finucane et al. 2015; Schaid et al. 2018). Of these, more than 90% of the variants were SNPs. In addition, the density of SNPs in lncRNA regions is similar to that in protein-coding regions. Some lncRNA intervals even have higher SNP densities than the genomic mean (Jin et al. 2011). SNP variants in lncRNA can affect mRNA expression through variable shear, localization, and stability of mRNA. Therefore, the association between lncRNA SNPs and phenotypes needs to be studied in depth. It has been shown that lncRNAs can influence complex traits at multiple levels of epigenetic regulation, transcriptional regulation, and post-transcriptional regulation (Zhang et al. 2018). To provide a comprehensive resource for linking lncRNAs to phenotypes. First, by carrying out RNA-seq analysis and sorting out the data of various non-coding region databases in RNAcentral and EVLncRNAs, we obtained a total of 203,391 LncRNA sequences. Precisely, 32,397, 32,192, 43,659, 8,741, 11,565, 25,884, 27,623, 12,577, 8,753 lncRNAs were obtained for *Zea mays* L., *Gossypium barbadense* L., *Triticum aestivum* L., *Lycopersicon esculentum* Mille, *Oryza sativa* L., *Hordeum vulgare* L., *Sorghum Bicolor* L., *Glycine max* L., and *Cucumis sativus* L., respectively. And based on the standard screening process, we integrated 2,000 important agronomic traits and 120,271 SNPs that have a significant effect on the phenotype of the nine species from the 421 articles. Among them, *Oryza sativa* L. and *Zea mays* L. have 764 and 573 traits, respectively, which account for 66.85% of all traits, while *Gossypium barbadense* L. has the least traits, which account for 0.5%. Meanwhile, 68,862 lncRNA sequences that can regulate important agronomic traits were predicted (Table 1).

**Table1:** Detail information about LncPheDB

Species	Phenotype	Var	Publications	LncRNAs	lncRNAs (Phenotype)	Version
*Zea mays* L.	573	71,058	151	32,397	28,164	B73_RefGen_v4
*Gossypium barbadense* L.	10	111	2	32,192	813	GCA_008761655.1
*Triticum aestivum* L.	50	755	11	43,659	4773	refseqv1.0
*Lycopersicon esculentum* Mille	132	787	9	8741	1212	ITAG4.0
*Oryza sativa* L.	764	23,690	117	11,565	8384	MSU_osa1r7
*Hordeum vulgare* L.	17	750	6	25,884	5508	version.1.0
*Sorghum Bicolor* L.	250	17,855	57	27,623	16,431	GCF_000003195.3
*Glycine max* L.	193	5129	66	12,577	3273	GCF_000004515.5
*Cucumis sativus* L.	11	136	2	8753	304	GCF_000004075.3

**Fig. 2:**
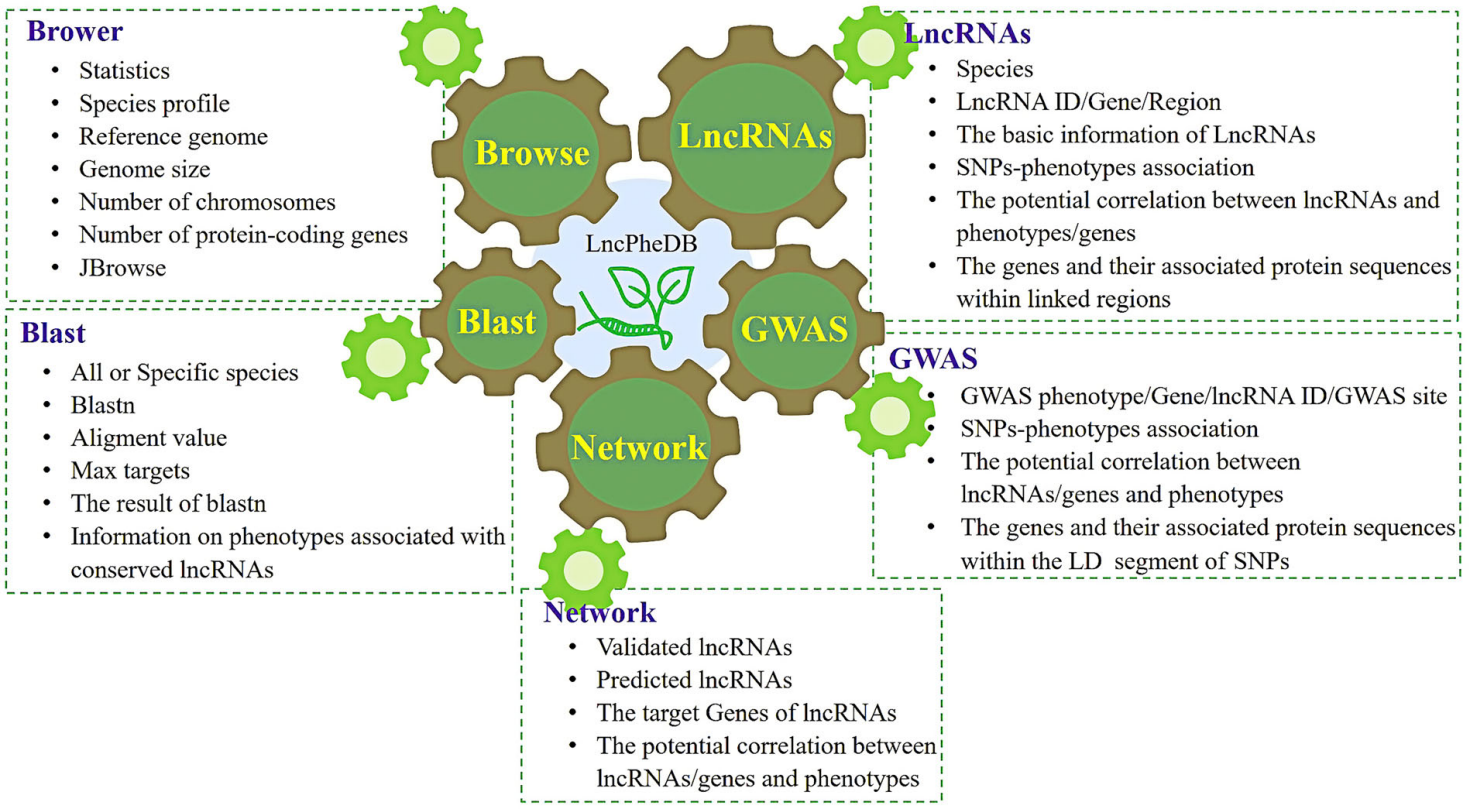
Database contents and functions of LncPheDB

In addition, to make it easier and more efficient for users to use the data. We provide a web service interface-LncPheDB. LncPheDB provides a user-friendly interface, a visual platform and a variety of search options. The LncPheDB database mainly provides the reference genome information of nine species (the size of the reference genome, number of chromosomes, and number of protein-coding genes). Basic information regarding all lncRNAs and phenotype-related lncRNAs (e. g. species, lncRNA identity (ID), chromosome, start site, termination site, and positive and negative chain), as well as basic information of GWAS results (e. g. GWAS phenotypic traits, location of peak in genome, and *P*-value) is provided. Furthermore, LncPheDB also provides functional information on genes associated with lncRNAs and protein sequence information of genes in various species (by searching the SWISS-PROT database), and the regulatory network information of lncRNAs related to phenotypes (Fig. 2).

LncPheDB provides two search engines: the lncRNA search engine and the GWAS search engine. The lncRNA module provides comprehensive lncRNA-phenotype correlation data in each species, which are created in the form of columns into tables. Each correlation data mainly includes phenotype-related lncRNA ID, species, chromosome position, lncRNA initiation and termination sites, Positive and negative chains, regulated phenotype, Peak Position, *P*-value of phenotype-SNP correlation, mapped genes, and sequence of mapped genes. In this module, we merge adjacent significant SNPs whose distance is less than the species LD into a single association signal based on the LD decay of each species. The SNP with the minimum *P* value in a signal region was considered to be the lead SNP. Finally, the related lncRNA and mRNA were predicted according to the LD of each species. This module focuses on exploring the linkage among SNPs and the linkage between SNPs and lncRNA or mRNA. There are also more phenotypes highlighted in this module, such as: the SNPs 201,770,002 (*P* = 3.65E-59), 201,770,047 (*P* = 4.97E-07), and 201,770,048 (*P* = 3.65E-59) located on chromosome 2 are significantly associated with maize leaves, and the SNPs is located within the lncRNA URS0000D75A41_4577.4871 (201,769,823–201,770,124). So we speculate that lncRNA URS0000D75A41_4577.4871 may be associated with maize leaves. In addition, for lncRNAs of interest, users can use our database for in-depth exploration. For instance, for maize lncRNA EL0549, after selecting the maize species, if you enter lncRNA EL0549 and click “search”, you can easily find information regarding the position of lncRNA EL0549, relevant GWAS information, and the information that EL0549 regulates maize’s flour fiber content, proline content, breakdown viscosity, flour fiber content, flour protein content, ear infructescence position, and maize kernels. To further determine the biological processes between lncRNA and traits, such as maize entrainment, protein content, and fiber concentration, among others, Users can click “Function” to view the functional information of genes associated with lncRNAs. Meanwhile, users can also click “Sequence” to view the protein sequence of genes (Supplemental Fig. S1). By phenotype, lncRNA/Gene ID or GWAS locus input**,** the GWAS module can be used to obtain phenotype-associated genes or lncRNAs for each species, genome-wide variant loci significantly associated with phenotypes, correlation *P* values, etc. The correlation data for this module are mainly obtained based on the amplification of individual variant loci, emphasizing the relative position between the variant loci and the lncRNA or gene. In the GWAS module, users can explore the phenotypes of their interest. For instance, the keyword “100 grain weight” can be used for maize (Supplemental Fig. S2). All search results can be downloaded in the form of a list. The combination of this lncRNA module and the GWAS module allows for a more comprehensive genome-wide prediction of phenotypic traits that may be regulated by lncRNAs or gene. Meanwhile, we also added the JBrowse genome browser, which allows users to intuitively search for the relative position distribution of lncRNAs and genes on chromosomes.

To study the sequence similarity, we designed a Blast tool (version 2.12). By searching specific species in the whole database, the BLAST service enables users to search for similar lncRNA sequences. In the BLAST results, users can directly view the phenotypic traits related to lncRNAs with similar fragments by clicking the “Click here to search LncRNA: lncRNA ID” tab. To enable users to view LncRNA and its regulated target genes clearly and concisely, we predicted the target genes of known and predicted lncRNAs by psRobot (Wu et al. 2012), psMimic (Wu et al. 2013) and IntaRNA (Mann et al. 2017), which were presented in the form of regulatory networks, marked them with different colors, and set three buttons, which allow users to hide corresponding genes by clicking the corresponding buttons. In addition to downloading the information from the corresponding search page, users can also download the reference genome information for each species, the lncRNA fasta sequence files, lncRNA Potential Encoding File, lncRNA Expression File and the GFF files for database construction via the download page. Moreover, users can also download the GWAS information file (such as associated phenotypic information, SNP, p-value, and information about studies) and the gene GFF file of each species.

## Discussion

With the development of sequencing technology in the past few years, a large number of lncRNAs have been identified and great progress has been made in the study of lncRNAs in plants. However, compared with the lncRNAs in animals and humans, there is a very limited understanding of lncRNAs in plants, especially in terms of the mechanism of lncRNAs in regulating important agronomic traits and affecting the yield and quality of model plants (Heo et al. 2013; Liu et al. 2012; Mann et al. 2017; Xiao et al. 2009; Yang et al. 2014). With the deepening of research, some well-annotated databases, such as PLncDB V2.0 (Jin et al., 2011) and GREENC (Gallart et al. 2016), have given comprehensive annotations to some basic information of lncRNAs, such as the position and sequence. Researchers have shifted their focus from identifying new lncRNAs to the functional research of lncRNAs. In recent years, researchers have investigated the functions of lncRNAs in plants. However, at present, the identified lncRNAs whose regulatory mechanism has been clarified are less than 1% (Quek et al. 2015). In addition, the research results of some lncRNAs provide a low reference value for the study of other lncRNAs due to the differences in types and functions of lncRNAs, which affect gene expression in a wide range at different levels. Therefore, researchers’ understanding and research on lncRNAs are limited. At present, it is imperative to use a genome-wide database to investigate the relationship between lncRNAs and phenotypes and explore the potential regulatory mechanism of lncRNAs.

Compared with other plant lncRNA databases, LncPheDB focuses on exploring data resources about lncRNA-regulated phenotypes. Using standardized screening criteria, LncPheDB manually sorted a total of 203,391 lncRNA sequences, 2000 phenotypes, and 120,271 SNPs. Finally, it listed 68,862 lncRNA sequences that are associated with agronomic traits. And according to the study. The lncRNA osa-eTM160 (Osa-eTM160 is a 688 bp long lncRNA transcribed between LOC_Os03g12815 and LOC_Os03g12820 of rice chromosome 3) in rice has a role in regulating rice fertility and seed size by competitively binding OsmiR160 with OsARF18. However, the potential regulatory significance of lncRNA URS00008EDDE3_39947.4350 (also known as osa-eTM160) on rice seed fertility, days to flowering, seed weight, arsenic accumulation, germination rate and grain Mn concentration is predicted in our database, which further confirms the significance of our database. Moreover, users can use the lncRNA sequences they are investigating to conduct a BLAST comparison with all species in the data resource to identify the conservative lncRNA-regulated phenotypes. Furthermore, LncPheDB also provides users with convenient browsing and search services. Thus, users can search lncRNAs correlation from various aspects, such as Gene ID, LncRNA ID, genome position, SNP, and phenotype. To help users explore the potential molecular regulatory mechanism of lncRNAs in complex traits, we summarized and sorted out the target gene prediction of lncRNAs and visually displayed it in the form of a regulation network. Users can hide or display the corresponding data by clicking different buttons.

As a future perspective, by focusing on the study of data resources regarding lncRNA-regulated phenotypes, we will add more lncRNA-related phenotypes for more species. In addition, since we found that the number of relevant studies was unexpectedly large when collecting and sorting out data, we will sort out more data regarding lncRNA-regulated phenotypes with clear regulatory mechanisms and predictions from existing studies and timely update the data resources. To further clarify the regulatory mechanism of lncRNAs, we will add more sequence information of miRNAs that are complementary to lncRNAs and increase the tissue-specific expression information of lncRNAs. Meanwhile, to enrich the transcriptome information of rice, we will add relevant transcriptome data in our research to facilitate scientific research and utilization. Notwithstanding, we also encourage all researchers to submit their relevant studies via the contact page. We believe that LncPheDB will provide assistance for the study of the functions of lncRNAs.

## Supplementary Information

Below is the link to the electronic supplementary material.

Supplemental Fig. S1 An example of searching from the lncRNA module. (A) Browse the reference genome of Maize. (B) Search for potentially relevant phenotypes of this lncRNA from the “lncRNA” module using lncRNA ID “EL0549”. (C) Related genes and their protein sequences within linked regions. (D) The regulatory network of lncRNA “EL0549”. (E) Blast in all resources was performed with lncRNA EL0549 sequence. (F) In all species, related lncRNAs that are conserved with the sequence of lncRNA EL0549. (G) Important agronomic traits regulated by related conserved lncRNAs (JPG 4709 KB)

Supplemental Fig. S2 An example of searching from the GWAS module. (A) In the GWAS module, select the species “Zea mays L.” and search using the keyword “100-grain weight”. (B) Found lncRNAs that have potential regulatory effects with the phenotype “100-grain weigh” in maize (C) Related genes in the LD segment of the mutation site “157,200,591” (D) Regulatory network of lncRNA “ZMAY_LNC002580.1” potentially correlated with phenotype “100-grain weight”. (E) Find lncRNAs that are conserved with lncRNA “ZMAY_LNC002580.1” in all resources. (F) The potential regulatory mechanism of the conserved lncRNA “ZMAY_LNC002580.1” (JPG 6082 KB)

Supplemental Table S1 Sources of RNA-Seq Information (XLS 1107 KB)

Supplemental Table S2 The basic information of GWAS articles (XLS 155 KB)

Supplemental Table S3 The degree of linkage disequilibrium information of each species (XLS 20 KB)

## Data Availability

LncPheDB is freely available at https://www.lncphedb.com/.

## Code availability

This study involves database building code.
