# Supplementary figures and images for: LncPheDB: a genome-wide lncRNAs regulated phenotypes database in plants
Source: aBIOTECH. 2022 Oct 5;3(3):169–77. doi: 10.1007/s42994-022-00084-3 (PMC9590470; doi:10.1007/s42994-022-00084-3)

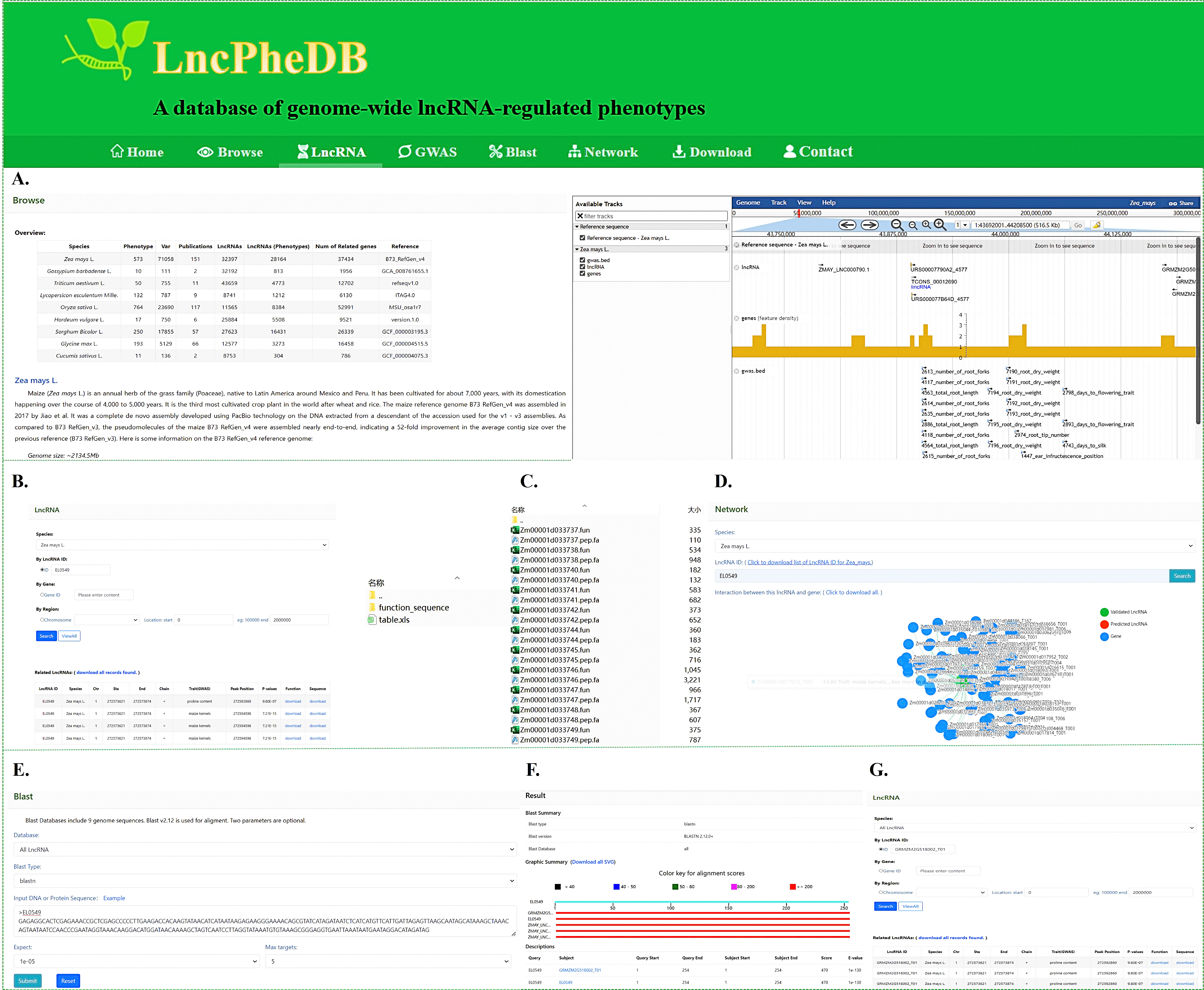

Supplement: Supplementary file 1 — Supplemental Fig. S1 An example of searching from the lncRNA module. (A) Browse the reference genome of Maize. (B) Search for potentially relevant phenotypes of this lncRNA from the “lncRNA” module using lncRNA ID “EL0549”. (C) Related genes and their protein sequences within linked regions. (D) The regulatory network of lncRNA “EL0549”. (E) Blast in all resources was performed with lncRNA EL0549 sequence. (F) In all species, related lncRNAs that are conserved with the sequence of lncRNA EL0549. (G) Important agronomic traits regulated by related conserved lncRNAs (JPG 4709 KB) [file 42994_2022_84_MOESM1_ESM.jpg]

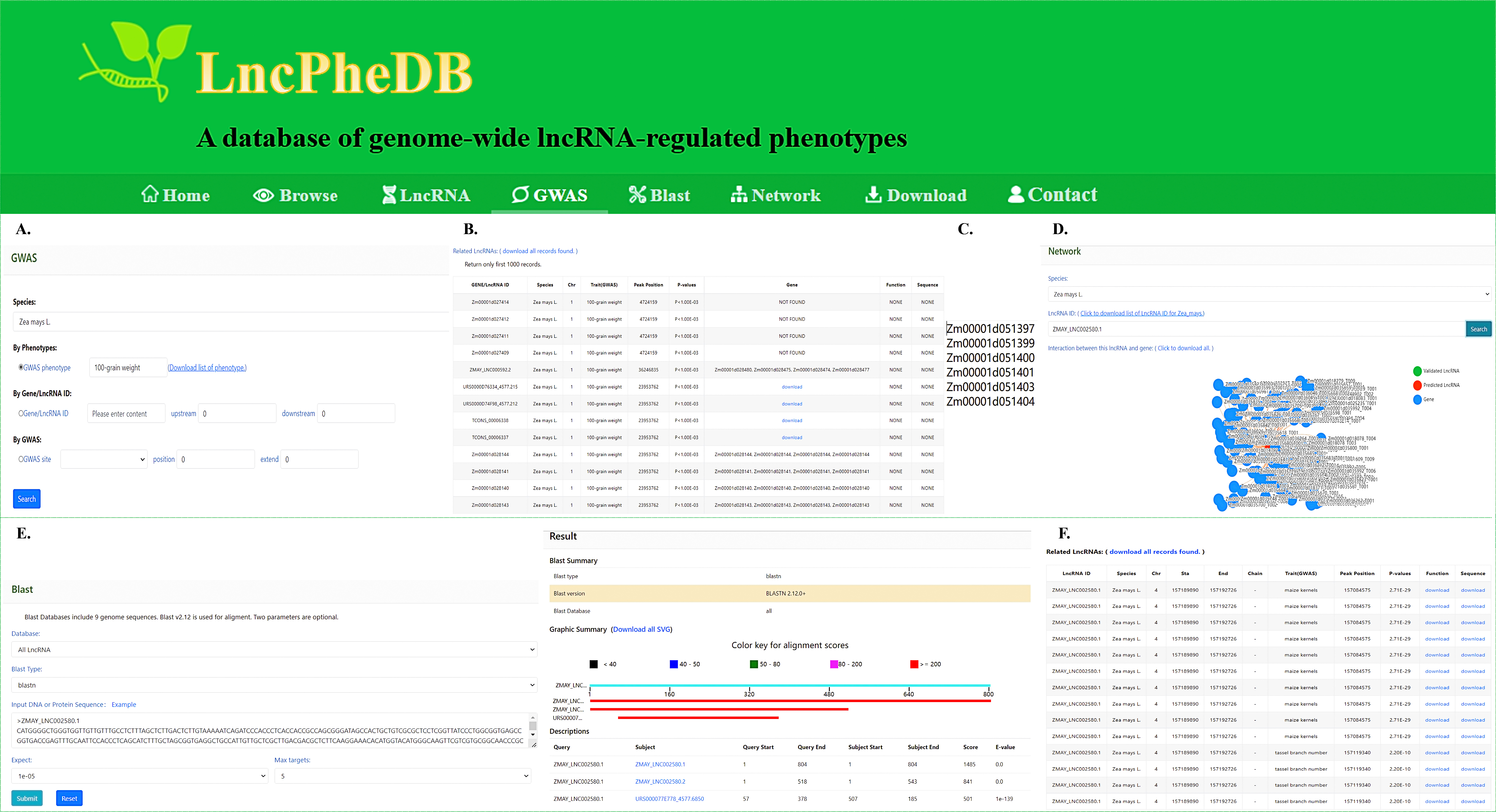

Supplement: Supplementary file 2 — Supplemental Fig. S2 An example of searching from the GWAS module. (A) In the GWAS module, select the species “Zea mays L.” and search using the keyword “100-grain weight”. (B) Found lncRNAs that have potential regulatory effects with the phenotype “100-grain weigh” in maize (C) Related genes in the LD segment of the mutation site “157,200,591” (D) Regulatory network of lncRNA “ZMAY_LNC002580.1” potentially correlated with phenotype “100-grain weight”. (E) Find lncRNAs that are conserved with lncRNA “ZMAY_LNC002580.1” in all resources. (F) The potential regulatory mechanism of the conserved lncRNA “ZMAY_LNC002580.1” (JPG 6082 KB) [file 42994_2022_84_MOESM2_ESM.jpg]
